# Retracted: Long Noncoding RNA KIAA0125 Potentiates Cell Migration and Invasion in Gallbladder Cancer

**DOI:** 10.1155/2017/3471417

**Published:** 2017-08-27

**Authors:** BioMed Research International

BioMed Research International has retracted the article titled “Long Noncoding RNA KIAA0125 Potentiates Cell Migration and Invasion in Gallbladder Cancer” [1]. This article is one of a series of very similar articles on shRNA and cancer cell lines identified by Byrne and Labbé [2]; the intertextual distance between this article and another of the series [3] is lower than expected by chance. The following concerns were found:

- The supposed nontargeting control shRNA sequence, 5 GCGGAGGGTTTGAAAGAATATCTCGAGATATTCTTTCAAACCCTCCGCTTTTTT-3, targets TPD52L2 (NM_199360). The same sequence was used as a nontargeting control in other articles identified by Byrne and Labbé. The authors say resequencing showed that the nontargeting sequence plasmid they bought is actually an empty vector.
- There is duplication between panels in Figure 5(a) (between the top left and top right panels and between the bottom left and center left panels), which the authors say was due to carelessness.
- The control panels of Figures 2(a) and 3(a) in this article are the same as in Figures 1(a) and 3(a), respectively, in the authors' article on Linc-ITGB1 [4], which was not cited. The authors said knockdown of KIAA0125 and Linc-ITGB1 expression by RNAi was performed simultaneously, so they shared the same controls.
